# Late-Onset Cervical Pseudomeningocele Following Ossification of the Posterior Longitudinal Ligament Surgery Successfully Treated With a Lumboperitoneal Shunt

**DOI:** 10.7759/cureus.30744

**Published:** 2022-10-27

**Authors:** Christos Tzerefos, Kostas Paterakis, Dimos Bouramas, George Fotakopoulos, Alexandros Brotis, Kostas Fountas

**Affiliations:** 1 Neurosurgery, General University Hospital of Larissa, Larissa, GRC; 2 Neurosurgery, Athens Bioclinic Hospital, Athens, GRC; 3 Neurosurgery, University of Thessaly, Larissa, GRC

**Keywords:** posterior longitudinal ligament, spine, spine surgery, pseudomeningocele, lumboperitoneal shunt

## Abstract

Pseudomeningocele (PMC) is a rare complication of anterior cervical procedures resulting in pain, headaches, nerve root entrapment, and in rare cases, spinal cord compression. Here we present a 57-year-old male with increasing myelopathy due to late-onset PMC that developed two years following a 360-degree cervical surgery for ossification of the posterior longitudinal ligament (OPLL). In this case, the PMC was successfully treated with a lumboperitoneal shunt.

A 57-year-old male presented with worsening symptoms and signs of cervical myelopathy. He had undergone a multilevel anterior corpectomy/fusion (ACCF), along with posterior fusion, two years earlier for severe ossification of the posterior longitudinal ligament (OPLL). Now presenting with increased myelopathy, his cervical spine MRI demonstrated a PMC in the perivertebral space, extending to and compressing the anterior cervical cord. Following a lumboperitoneal shunt insertion, the patient's myelopathy resolved.

Acute, subacute, or chronic postoperative cervical pseudomeningoceles (PMC) may be readily managed with a lumboperitoneal shunt insertion.

## Introduction

Pseudomeningocele (PMC) is an abnormal collection of cerebrospinal fluid (CSF) that creates communication between the subarachnoid space in the spinal canal and the paraspinal soft tissue. Rarely, PMCs result in pain, headache, nerve root entrapment, and spinal cord compression [1].

Most reports of PMCs occur following posterior surgery, while only a few cases have been described following anterior procedures [1-4]. However, PMCs may appear in ossification of the posterior longitudinal ligament (OPLL) surgery, with a reported incidence as high as 100% after surgery for OPLL [5-9]. Whereas asymptomatic PMCs can be conservatively managed, those contributing to symptoms warrant immediate treatment, consisting of shunting and/or direct surgical repair of the dural defect [7].

Here, we present a 57-year-old male developing a PMC after a 360º cervical procedure for OPLL that was successfully treated with a lumboperitoneal shunt insertion.

## Case presentation

**Figure 1:**
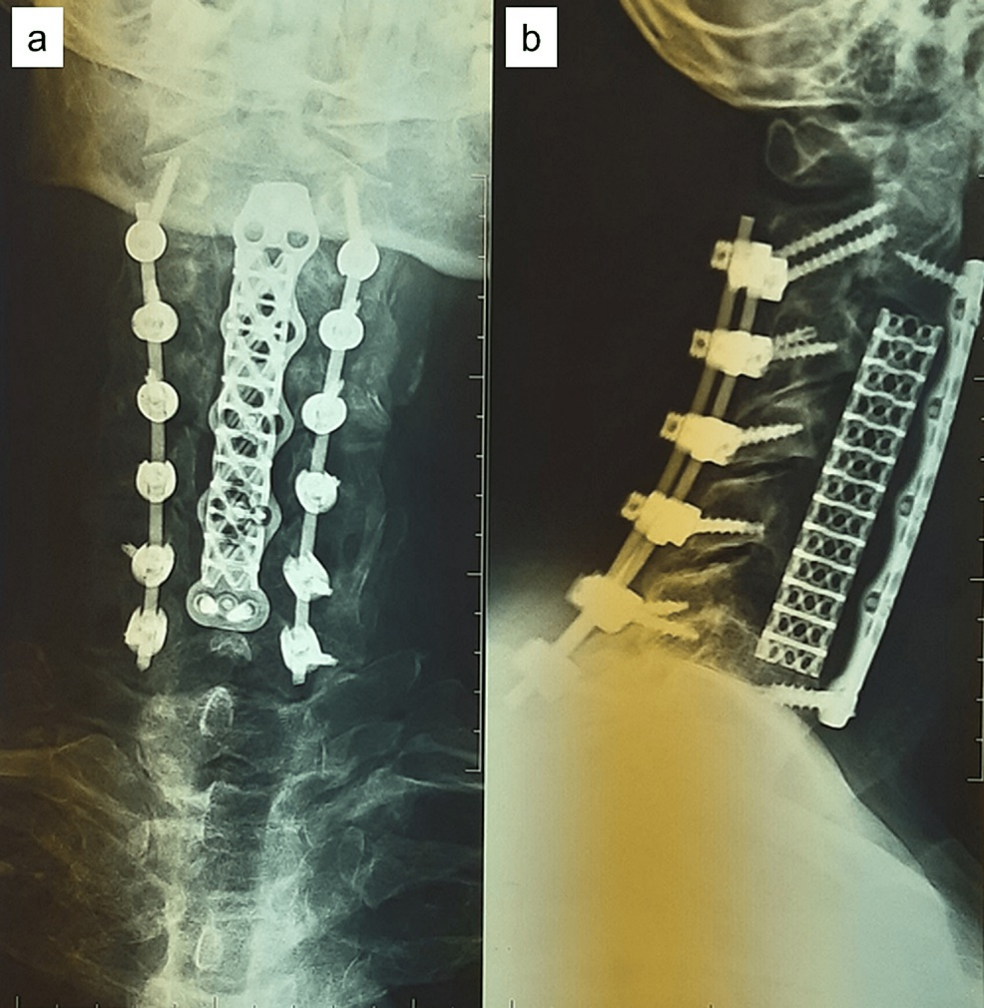
Postoperative X-ray Cervical plain radiographs show a titanium mesh at the site of corpectomy C3-C5 secured by an anterior plate as well as a posterior pedicle, lateral mass screws and rods C2-C7: (a) anteroposterior, (b) lateral

A 57-year-old male with cervical myelopathy (the patient was able to perform fine movements with difficulty and walk on a flat floor only if assisted; however, the patient reported only minimal sensory loss and normal micturition (modified Japanese Orthopaedic Association score of 11)) due to multilevel OPLL had undergone a multilevel anterior cervical corpectomy and fusion from C2-C6 (corpectomies of C3, C4, C5), with posterior pedicle and lateral mass screw/rod fixation from C2 to C7, under intraoperative neural monitoring (Figure 1).

A dural tear was noticed during anterior decompression, treated with Tisseel (Baxter, Deerfield, Illinois) and TachoSil (Corza Medical, Westwood, Massachusetts) applications. We considered that the performed corpectomies and the ample space between the anteriorly placed mesh cage and the dura would allow expansion of the patch with no cord compression. No further CSF leak was noticed, and after the insertion of a subcutaneous gravity drain, the surgical wound was closed in anatomical layers. The postoperative course was uneventful, and his symptoms improved. The inserted drain was removed on the second postoperative day. Because of the improvement in his clinical condition, no postoperative MRI was performed. Postoperatively, the patient's hand function and walking improved, with a modified Japanese Orthopaedic Association (m-JOA) score of 13, whereas there were no clinical manifestations of intracranial hypotension, such as headache and nausea, and vomiting.

**Figure 2:**
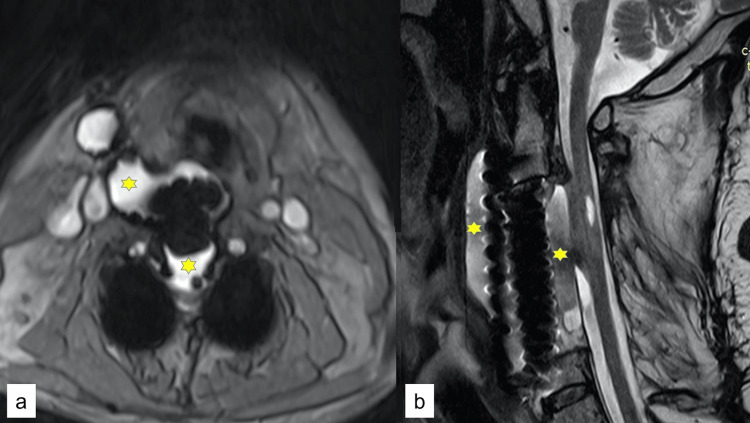
Pre-operative MRI Pre-operative axial (a) and sagittal (b) T2-weighted images showing cerebrospinal fluid in the perivertebral space (stars)

One year later, the patient returned with a recurrence of his symptoms, along with a minimal dysphagia. The repeat MR T2 study was consistent with an anterior PMC that extended from the perivertebral space into the anterior epidural space resulting in cord compression (Figure 2).

**Figure 3:**
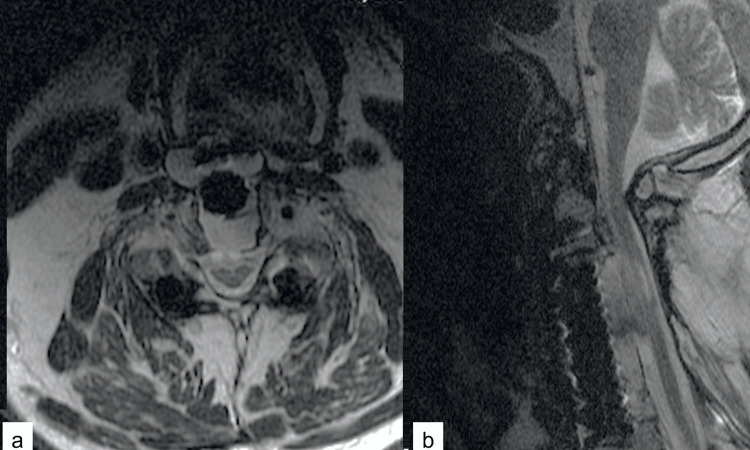
Postoperative MRI Postoperative axial (a) and sagittal (b) T2-weighted images show a decrease in the cerebrospinal fluid collection, and an increase in spinal cord diameter, seen better on the axial image

The patient refused surgical treatment offered at that time. Two years later, due to the enlarging neck mass, other symptoms, such as neck pain, dysphagia, and hoarseness, led to further worsening of his clinical condition, and he agreed to have surgical treatment. Initial lumbar drainage of 30 ml of CSF via a spinal tap improved his symptoms. Therefore, a lumboperitoneal (LP) shunt was inserted, and one month after the procedure, his symptoms started to improve. A postoperative MRI showed that the fusion was successful, and the fluid collection decreased without any effect on the arthrodesis (Figure 3). Two years later, the patient remains neurologically unchanged.

## Discussion

Cerebrospinal fluid leak and collections of the retropharyngeal space constitute a rare complication of anterior cervical spine surgery ranging from 0.03 to 7.7 % [8]. Ailon et al. [1], in their multicenter cohort study, presented an incidence of PMCs of less than 0.1% in a population of 17,625 patients. Although many cases result from trauma, PMCs may occur more frequently following anterior cervical OPLL surgery, with the reported dural tear rate varying from 4.3% to 32% [7, 9-12]. The incidence of PMCs in OPLL surgery can be as high as 7.7% [5, 6, 13, 14]. An actual meningocele forms when the arachnoid membrane remains intact [4, 15].

The development of a PMC may be considered a mechanical process. Lesion size and time of presence depend on the size of the dural defect, the pressure of the spinal fluid, and the resistance of the surrounding soft tissues. This explains why PMCs rarely appear in the cervical spine and more frequently in the lumbar spine [16]. In dural-arachnoid tears, extravasation of CSF will form a PMC, a CSF pseudocyst lined by fibrous tissue. A true meningocele forms when the arachnoid membrane remains intact [4,15].

A pseudomeningocele can cause symptoms related to CSF hypotension, such as blurry vision, dizziness, postural headaches, diplopia, headaches, tinnitus, back pain, radicular symptoms, nausea, and vomiting, as well as neurologic deficit mainly resulting from herniation of neural tissue through the dural defect [17, 18]. In our case, although there was no herniation of the spinal cord, the patient presented with a worsening of his clinical condition.

In asymptomatic PMC cases, the literature has proposed a conservative approach on certain occasions [1, 4]. For patients with acute, subacute, or chronic PMC, or persistent CSF fistulas following OPLL surgery, treatment options should include reoperation for repairing the underlying dural defect and eliminate the CSF leak and/or temporary lumbar drain insertion, with conversion to permanent lumboperitoneal shunt, and/or wound peritoneal shunt [1, 3, 19]. It has to be noted that the use of any fibrin sealant carries the risk of swelling and subsequent cord compression [20]. Although it was used in our case due to the previously performed adequate cord decompression and the existence of ample epidural space, the risk of swelling and cord compression needs to be seriously taken into consideration. Reoperation and surgical repair are not always feasible because of the implantation of cages and plates during the initial surgery, as in our current case. Moreover, using a lumbar drain or repeated lumbar punctures may well work in the majority of acutely presenting cases [1].

## Conclusions

Pseudomeningocele is a rare complication of anterior cervical surgery in patients with OPLL. Although a conservative approach may be preferred in some cases, for patients with acute, subacute, and chronic symptoms/signs of persistent anterior cervical CSF leaks, lumbar drain placement followed by lumboperitoneal shunting and/or wound-peritoneal shunting and if there is a clinical improvement then should be considered. The ultimate treatment should be tailored to the patient's clinical and imaging features. Nevertheless, further studies are required to establish the optimal treatment for postoperative pseudomeningocele after anterior spine surgery.
